# New York Letter

**Published:** 1893-02

**Authors:** 


### NEW YORK LETTER.

NEW YORK, January 27th, 1893.

The epidemic of typhus fever now existing here does not seem to be of a very virulent type; in fact, the mortality thus far has been very low. From December 1st to January 17th, there were 144 cases reported, with only forty-one deaths—a mortality of only a small fraction over 28.6 per cent. Though every thing that is possible is being done to eradicate the disease, there are still a few cases appearing here and there in the lower tenement-house districts. Not a single case has been found outside of these crowded and filthy districts, and, thanks to the persistent efforts of the health officers, (perhaps aided to some extent by the unusually cold weather we have been blessed with), but few cases have developed in any one house. While the cold may have had something to do with the destruction of the contagion, it certainly fostered the disease by causing the crowding together of the exposed in these dirty and ill ventilated quarters.

Whenever a case appears, it is immediately removed to the hospital set apart for patients suffering from this disease; the quarters are thoroughly disinfected, and other occupants of the same house are carefully guarded until all danger from their spreading the disease is passed. Though most of the cases can be traced to some source of infection, still a few cases have sprung up in which it has been quite impossible to find where or how they became infected, and how to disinfect so as to prevent the appearance of such cases is a hard question for the health officers to answer. For instance, a tramp who has been sleeping in station houses, or any where else he can find a resting place, is suddenly taken with the disease. When he became infected, or where he has been since, it is almost impossible to say, and quite out of the question to think of disinfecting every saloon, dive or dwelling house he may have found his way into. Notwithstanding such cases floating around at times, the disease is becoming less and less frequent, and doubtless will soon be extinct in this country until a fresh ship-load is imported.

At a meeting of the County Medical Society, Dr. T. A. Emmet read an interesting and instructive paper on "The Means of Success in Gynecological Plastic Surgery." He thought that while the craze for abdominal surgery had developed some very expert laparotomists in this country, the skill necessary for plastic work had almost been lost. There is not only lack of dexterity in operating, but a lack of knowledge of what is to be accomplished, and many failures are the natural result. He dwelt upon the idea that only a single plastic operation should be undertaken at one sitting, and thought that many failures were due to the fact that two or three operations were performed at the same time. When only a single operation is performed, there is much more likelihood of primary union which is essential to success by avoiding granulation and scar tissue. He said the chief cause for the symptoms in laceration of the floor of the pelvis is lack of support for the blood vessels. Both the butterfly and trefoil operation, which Dr. Emmet devised some time ago, and his more recent modification, would repair the damage. But the dashboard-like obstruction put up at the vaginal outlet by some operators is a failure. While he had always been able to repair any injury of the female genital organs which he had met with, produced by the usual causes, those left by poor surgery had taxed him to the uttermost.

He attributes his success in plastic surgery about the female genitals to the use of the silver wire suture, which he claims acts both as a suture and a splint. Nothing but silver wire should be used in any plastic operations about the vagina, is the rule he follows, and his wonderful success should lead others to do likewise.

Dr. Munde classed the elements of success in plastic surgery under a few heads : 1. Proper selection of cases ; (2) selection of the proper time for operation ; (3) proper preparation of the parts; (4) perfect asepsis involving strict cleanliness, but avoidance of strong germicides; (5) rest, yet early movement of the bowels; (6) he could not agree with Dr. Emmet that the silver wire suture was so universally necessary or useful. He employed silk for operations on the anterior vaginal wall, and silkworm gut at the perineum. He disagreed with Dr. Emmet in condemning several operations at a time, as he had saved many women much time, pain and expense by performing several operations at one sitting.

At the meeting of the Section on Surgery at the Academy, Dr. Bryant presented a man in which a large iliac aneurism had been cured by needling after McEwen's method. He was thirty-five years of age; had had a chancre fifteen years ago. About two years ago, while lifting a heavy weight supported against the iliac region, he experienced a tearing sensation which was soon followed by a pulsating tumor above Poupart's ligament. The man had been treated by other surgeons before coming to Dr. Bryant, one of whom put "something into the tumor," probably a wire; weight had also been applied, but there was no evidence of the tumors becoming any smaller or the pain any the less. When he came under Dr. Bryant's care about six months ago, he was suffering severe pains. The tumor was apparently connected with the right iliac artery. He had long needles made, varying in diameter from one-half to one millimetre. The smaller were introduced at first, and with them he was unable to tell when the opposite side of the tumor was reached. Two of the tumors were left in twenty-four hours without appreciable change. The larger needles were introduced six days later and left in forty-eight hours. Within a week the bruit lessened, and disappeared entirely in two weeks, and soon there was no pulsation or pain. When presented, the tumor was formidable in size, but hard, and Dr. Bryant thought he could make out a slight pulsation. There was some question raised as to whether the clot formed was of the red or white variety, but so long as the symptoms are relieved, it seems to me that it should make little difference whether the clot formed be of one variety or another.

An interesting case was operated on by Dr. Gerster at the Mount Sinai Hospital last week, and one which should warn practitioners against treating diseases of the rectum without making an examination. The patient was a young man who gave a history of some rectal trouble which had been treated, without an examination, as hemorrhoids by ointments, etc., for some time. On examination it immediately became evident that there was a large number of polypi in the rectum. When he was anesthetized and the sphincters stretched, the rectum was found to be simply packed with polypi, varying in size from that of a peach pit to a small shot, and extending as far up the organ as the finger could reach. As many of them as could be reached were removed by ligation. About every two or three weeks the patient will again be anesthetized and any new growths removed. This treatment will have to be continued for a long time, as the very small tumors will have to grow to some size before they can be ligated. Too many practitioners are averse to putting their fingers in a patient's rectum and are willing to treat anything that gives a history of rectal trouble as "piles," and even then with an ointment that only lessens the patients sufferings for a time, and has no real effect on the hemorrhoids, if indeed it be hemorrhoids that is troubling the patient. Only a short time ago, I saw a patient in one of the clinics who was wearing an ointment of some astringent variety over an ischeo-rectal abcess. On being asked what he had that there for ; he said, "the doctor gave it to me to cure my piles," but the doctor had never made an examination, and the patient had not the first sign of a hemorrhoid, but was verging on septicemia from the absorption of pus from this abcess, which "the doctor" was treating with an astringent ointment. Many say the rectum is too filthy a place to examine, but if these men would examine a rectum after an anema, and then examine a leucorrheal vagina, which none of them seem to hesitate to do, they would see that the latter is by far the more disgusting organ of the two.

